# Trajectories of antidepressant use and characteristics associated with trajectory groups among young refugees and their Swedish-born peers with diagnosed common mental disorders—findings from the REMAIN study

**DOI:** 10.1007/s00127-021-02139-0

**Published:** 2021-07-23

**Authors:** S. Rahman, S. Filatova, L. Chen, E. Björkenstam, H. Taipale, E. Mittendorfer-Rutz

**Affiliations:** 1 Epidemiology of Psychiatric Conditions, Substance Use and Social Environment (EPICSS), Department of Global Public Health, Karolinska Institutet, SE-113 65Solnavägen 1E, Stockholm, Sweden; 2 Division of Insurance Medicine, Department of Clinical Neuroscience, Karolinska Institutet, Stockholm, Sweden; 3 Niuvanniemi Hospital, Kuopio, Finland

**Keywords:** Migration, Refugee, Antidepressant, Common mental disorders, Sick leave, Disability pension, REMAIN

## Abstract

**Purpose:**

This study aimed to (1) identify the trajectories of prescribed antidepressants in refugee youth and matched Swedish-born peers diagnosed with common mental disorder (CMD) and (2) characterize the trajectories according to sociodemographic and medical factors.

**Methods:**

The study population comprised 2,198 refugees and 12,199 Swedish-born individuals with both Swedish-born parents, aged 16–25 years in 2011, residing in Sweden and treated in specialised healthcare for CMD 2009–11. Group-based trajectory modelling was used to identify different trajectory groups of antidepressant use-based on annual defined daily dosages (DDDs). Multinomial logistic regression was applied to investigate the association of sociodemographic and medical characteristics with the identified trajectories. Nagelkerke pseudo-*R*^2^ values were estimated to evaluate the strength of these associations.

**Results:**

Four trajectory groups of antidepressant use among young refugees were identified with following proportions and DDD levels in 2011: ‘low constant’ (88%, < 100), ‘low increasing’ (2%, ≈710), ‘medium decreasing’ (8%, ≈170) and ‘high increasing’ (2%, ≈860). Similar trajectories, however, with different proportions were identified in Swedish-born: 67%, 7%, 21% and 5%, respectively. The most influential factors discriminating the trajectory groups among refugees were ‘duration of stay in Sweden’ (*R*^2^ = 0.013), comorbid ‘other mental disorders’ (*R*^2^ = 0.009) and ‘disability pension’ (*R*^2^ = 0.007), while ‘disability pension’ (*R*^2^ = 0.017), comorbid ‘other mental disorders’ (*R*^2^ = 0.008) and ‘educational level’ (*R*^2^ = 0.008) were the most important determinants discriminating trajectory groups among Swedish-born youth.

**Conclusion:**

The lower use of antidepressants in refugees with CMDs compared to their Swedish-born counterparts warrants health literacy programs for refugees and training in transcultural psychiatry for healthcare professionals.

## Introduction

Several European countries, including Sweden, have experienced the immigration of a growing number or refugee minors/youth [1, 2]. A considerable proportion of these refugee minors/youth have been exposed to traumatic events in their country of origin or during flight to their host country, putting issues of public mental health on the agenda [3]. Mental ill-health can range from psychological distress to diagnosed disorders. Most frequently occurring are common mental disorders (CMDs), including depressive, anxiety and post-traumatic stress disorder (PTSD). These disorders are characterised by an early age of onset, recurrent episodes and emerging comorbidity [4]. Among refugees, children and adolescents are often considered as the most vulnerable group as they experience the stressors of forced migration during their formative years [5]. Also, difficulties in adaptation to the new host country, possible racial discrimination and social marginalization such as labour market marginalization, may lead to mental health problems in young refugees [6–8].

Although young refugees have an elevated risk of mental disorders, studies have shown that they tend to utilize psychiatric care and psychotropic drugs to a lower extent than their peers born in the host country [9–13]. This may be due to barriers in accessing healthcare in general and individual-level factors related to socio-cultural perceptions and attitudes, but also practical problems such as language difficulties when communicating with healthcare professionals [14]. Studies from the Nordic countries showed that refugees are less likely to be dispensed psychotropic drugs, including antidepressants than their native counterparts [10–13].

Refugee minors/youth with CMDs are a heterogeneous group; therefore, different medical and sociodemographic characteristics such as comorbidities and socio-economic status might be relevant in determining differences in the course of mental disorders and hereby the treatment [15, 16]. Also, social marginalisation such as labour market marginalisation and migration-related factors such as duration of residency in the host country are of importance [17, 18]. These factors are related to the process of acculturation in the host country and thereby may affect the likelihood of seeking healthcare [19, 20] and being compliant to treatment [21, 22].

The heterogeneity in these sociodemographic and medical characteristics may, however, not only affect the treatment initiation, but also its course. In order to study treatment in young refugees with CMDs, advanced models capable of capturing this variation in patterns/trajectories are warranted. Therefore, the study used group-based trajectory modelling. Such method can distinguish subgroups of young individuals with CMDs following distinct trajectories by both levels and trends of antidepressant use during the study period. This gives the possibility to identify trajectory groups and explain their group differences by other factors, e.g., sociodemographic, medical factors, etc. Additionally, antidepressants are prescribed in both primary and specialised healthcare and therefore can include the entire range of healthcare [23]. To the best of our knowledge, no previous study investigated the trajectories of antidepressant treatment in young refugees with CMDs over a time period, while also characterizing the different trajectory groups.

This study aimed to (1) identify the trajectories of prescribed antidepressants in refugee youth and matched Swedish-born peers with both Swedish-born parents diagnosed with CMD, (2) characterize the trajectories according to sociodemographic and medical factors and (3) compare these trajectories and characteristics between refugee youth and their counterparts born in Sweden.

## Methods

### Data sources, linkage of the nationwide registers

We used the unique (de-identified) Swedish personal identity number [24] to link information from several population-based registers. The Longitudinal Integration Database for Health Insurance and Labor Market Studies (LISA) contains data on sex, age, education, living area and family situation since 1990 [25]. The Longitudinal Database for Integration Studies (STATIV) register holds migration-related information, including reasons for settlement, e.g. refugee status [26]. The National Patient Register (NPR) includes information on inpatient and specialised outpatient care since 1987 and 2001, respectively [27, 28]. All diagnoses in NPR are coded according to the ICD-10 [29] since 1997. The Prescribed Drug Register (PDR) contains information on dispensed prescribed drugs to patients from entire Sweden since July 2005 [30]. Pharmaceuticals in PDR are grouped according to the Anatomical Therapeutic Chemical Classification system codes (ATC) [31]. Individual’s parental birth country was identified by linking families through the Multi-Generation Register [32], which contains all known relationships between children and parents (born 1932 or later) since 1961. Finally, the Cause of Death Register includes information on all deaths of Swedish residents since 1952 [33].

### Study population

The study base (*N* = 361,993) included young refugees aged 16–25 years, identified in the STATIV register during 2011, who resided in Sweden during 2009–11 (*n* = 60,333) and five Swedish-born individuals with both biological parents born in Sweden matched to each refugee on sex, age and living area, (*n* = 301,665). Due to missing data for different covariates five refugees were removed leaving 60,328 refugees in the study base. From this study base, young individuals who were treated at inpatient or specialised outpatient healthcare due to any CMD, alive and living in Sweden during 2009–11, were selected. The final study population, included 2198 refugees and 12,199 Swedish-born individuals.

### Measures

#### CMD definition

CMDs were defined by codes from the International Classification of Diseases version 10 (ICD -10 codes) and extracted from the NPR. The specific codes were F32–F33 (depressive disorders), F40–F42 (anxiety disorders), F43.1 (posttraumatic stress disorder) and all F43 but F43.1 (other stress-related mental disorders).

#### Refugee or Swedish-born status

The term ‘refugee’ was used to define an individual with a residence permit in Sweden based on the Geneva Convention of Refugees [34] or an individual who was granted residence permit due to ‘in need of protection’, ‘humanitarian grounds’ or through ‘family reunification to refugees’. These four categories are subsequently called ‘refugees’. Sensitivity analyses revealed the comparability of these groups regarding the trajectories of antidepressant use. An individual was defined as ‘Swedish-born’ if the birth country was Sweden for both the individual and both biological parents.

#### Exposure measure

Antidepressants were defined as ATC code N06A. We extracted the annual defined daily dosage (DDD) of prescribed antidepressants for the three studied years 2009–11 (Y1–Y3). Then we summed up DDDs of all antidepressants the individual was prescribed during each calendar year. Annual cumulative DDDs exceeding 1500 (around 4 DDDs) were deemed unusual (possibly due to special cases, large purchases before traveling abroad, or error in data) and truncated at a level of 1500.

#### Covariates

A range of sociodemographic and medical covariates were included. If not stated otherwise, the covariates were measured on December 31st, 2011. Sociodemographic included sex, age, educational level, living area and family situation. Additionally, factors related to labour market marginalisation (measured during 2009–11), considered as sickness absence and disability pension, as well as duration of stay in the host country (for refugees) were used. Categorisations of the variables are presented in Table 1. In Sweden anyone aged 19–29 years can be granted temporary disability pension due to disease or injury from the Social Insurance Agency, and those aged 30–64 years can receive permanent disability pension [35].

**Table 1:** Descriptive statistics of the study population, including refugees and Swedish-born individuals, aged 16–25 years, living in Sweden throughout 2009–11 with specialised healthcare use due to any common mental disorder during the same period (2009–11)

	All (*N* = 14,397)		Refugee (*n* = 2198)		Swedish-born (*n* = 12,199)	
	*N*	%	*n*	%	*n*	%
Sex^a^						
Female	8772	60.9	1169	53.2	7603	62.3
Male	5625	39.1	1029	46.8	4596	37.7
Age (in years)^a^						
16–19	2112	14.7	319	14.5	1793	14.7
20–25	12,285	85.3	1879	85.5	10,406	85.3
Education (in years)^a^						
0–9	5857	40.7	995	45.3	4862	39.8
10–12	6076	42.2	724	32.9	5352	43.9
13 or more	1962	13.6	229	10.4	1733	14.2
Missing	502	3.5	250	11.4	252	2.1
Living area^1,a^						
Large cities	6183	43.0	923	42.0	5260	43.1
Medium cities	5851	40.6	855	38.9	4996	41.0
Rural areas	2363	16.4	420	19.1	1943	15.9
Family situation^a^						
Married/cohabiting	882	6.1	269	12.2	613	5.0
Single^2^	9670	67.2	1494	68.0	8176	67.0
Living with parents, < 20 years old	3845	26.7	435	19.8	3410	28.0
Specialised healthcare use due to CMDs during 2009–11 (only ‘yes’ are mentioned)						
Due to depressive disorders^3^	6643	46.1	793	36.1	5850	48.0
Due to anxiety disorders^4^	7599	52.8	876	39.9	6723	55.1
Due to posttraumatic stress disorder^5^	594	4.1	313	14.2	281	2.3
Due to stress-related mental disorders^6^	2626	18.2	705	32.1	1921	15.7
Antidepressant^7^ prescriptions during 2009–11						
Yes	10,207	70.9	1097	49.9	9110	74.7
No	4190	29.1	1101	50.1	3089	25.3
Comorbid other mental disorders than CMDs^8^ during 2009–11						
Yes	5842	40.6	1566	71.3	6989	57.3
No	8555	59.4	632	28.7	5210	42.7
Comorbid somatic disorders^9^ during 2009–11						
Yes	9305	64.6	1513	68.8	7792	63.9
No	5092	35.4	685	31.2	4407	36.1
Sickness absence during 2009–11 (in net days)						
0	12,226	84.9	2045	93.0	10,181	83.5
1–90	1089	7.6	77	3.5	1012	8.3
> 90	1082	7.5	76	3.5	1006	8.2
Disability pension during 2009–11						
Yes	1514	10.5	152	6.9	1362	11.2
No	12,883	89.5	2046	93.1	10,837	88.8

^a^Measured on 31-December-2011

^1^Type of residential area: big cities—Stockholm, Gothenburg and, Malmö; medium-sized cities—cities with more than 90,000 inhabitants within 30 km distance from the city center; small cities/villages

^2^Single includes divorced/separated/widowed

^3^Depressive disorders: according to ICD v10 codes F32, F33

^4^Anxiety disorders: according to ICD v10 codes F40–F42

^5^Posttraumatic stress disorder: according to ICD v10 code F43.1

^6^Stress-related mental disorders: according to ICD v10 codes F43, except F43.1

^7^Antidepressants: according to ATC code N06a

^8^Other mental disorders than CMDs: according to ICD v10 codes F00–F99, except F32, F33, F40–F43

^9^Somatic disorders: according to ICD v10 codes all diagnoses other than F00–99, O00–99, P00–96, Q00–99, R00–99, U00–U85, V01–Y98 (except X60–84 and Y10–34)

Medical factors measured during 2009–11 comprised measures of psychiatric and somatic comorbidity recorded as either primary or secondary diagnoses at inpatient or specialised outpatient healthcare. Comorbid ‘other mental disorders than CMDs’ included any mental diagnosis except depressive, anxiety and stress-related disorders, defined by the ICD -10 codes F00-F99, except F32, F33 andF40–F43. In the multinomial regression, the comorbid mental disorders included personality disorder (F60–F69), attention deficit hyperactivity disorder (ADHD) (F90), substance use (F10–F19, except F17), suicidal behaviour (X60–X84, Y10–Y34) and ‘other mental disorders’. In addition to the healthcare use data, screening of ADHD and substance use were supplemented by prescription information using ATC codes during 2009–11. ADHD medication included N06BA01, N06BA02, N06BA04, N06BA07 and N06BA09, and substance use comprised N07BB01, N07BB03, N07BB04, N07BC01, N07BC51 and N07BC02. Thus, ‘other mental disorders’ included any mental diagnosis other than CMDs, personality disorders, ADHD, substance use and suicidal behaviour (ICD 10: F00-99, except F32, F33, F40-F43, F60-F69, F10-F16, F18-F19, X60-X84, Y10-Y34; no medication with ATC: N06BA01, N06BA02, N06BA04, N06BA07, N06BA09, N07BB01, N07BB03, N07BB04, N07BC01, N07BC51 and N07BC02).

‘Comorbid somatic disorders’ included all diagnoses other than mental, pregnancy and birth-related diagnoses, unspecified signs and symptoms, external causes of morbidity and mortality (except intentional self-harm and event of undetermined intent), codes for special purposes in ICD, codes for healthcare contacts in ICD (all diagnostic codes except F00–F99, O00-O99, P00–P96, Q00–QR99, R00–99, U00–U85, V01–Y98 (except X60–84 and Y10–34)). Information on prescriptions of antidiabetics (ATC code: A10) during 2009–11 was also used to identify comorbid diabetes.

Data were missing only on education status with 11.4% missing in refugees and 2.1% in Swedish-born. These missing values were considered as a separate categories. Categorisations of all covariates are presented in Table 1.

### Statistical analyses

We used group-based trajectory modelling to estimate trajectories of antidepressants among refugees and Swedish-born individuals with treated CMD. These models estimate changes in antidepressant use patterns over time in multiple subgroups within the cohort, create a regression model for each discrete group and assess proportions of individuals in each group [36]. We used the Bayesian information criterion (BIC) to test the best-fitted model related to the number of groups. Moreover, we considered the proportions of individuals in each group in order not to have too small groups. Therefore, we decided to limit to four trajectory groups of antidepressants’ use. Probabilities for an individual to be assigned to a specific trajectory group were calculated. The highest estimated probability was used to decide each individual’s group belonging. Côté et al. recommend that the average probability for individuals of a trajectory group should be ≥ 0.70 [37]. Such average probability for individuals in our cohort was around 0.85, indicating a very good fit.

Trajectory analyses were conducted separately for refugees and Swedish-born individuals. Differences in distribution of sociodemographic and medical factors in different trajectory groups were assessed by Chi^2^-test. Multinomial logistic regression, performed separately for refugees and Swedish-born, was conducted to determine the contribution of different covariates to describe different trajectory groups. The likelihood ratio tests were used to evaluate whether sociodemographic and medical factors were associated with type of trajectory group in the full model. The full models included all covariates similarly for refugees and Swedish-born. The estimates for ‘duration of stay in the host country (Sweden)’ were calculated only in refugees, as this is not applicable in Swedish-born. Moreover, Nagelkerke pseudo-*R*^2^ values were estimated to evaluate the strength of these associations. By consecutively excluding and re-including each factor from the full model, we calculated differences in pseudo-*R*^2^ for each factor in order to examine the contribution of a given factor to the full model.

Data processing was performed using SAS version 9.4 (SAS-based procedure ‘Traj’ [38] and SPSS version 22.0 (Chi^2^-test and multinomial logistic regression).

#### Sensitivity analyses

In order to determine the comparability of all four sub-groups of the ‘refugee’, we conducted sensitivity analysis by including and excluding each sub-group one at a time to the ‘refugees defined by Geneva convention’, i.e., ‘in need of protection’, ‘humanitarian grounds’ or through ‘family reunification to refugees’ in the ‘refugee’ group, which showed similar trajectory groups of antidepressant use.

Trajectories of antidepressant use may differ between refugees and the Swedish-born population due to the differences in the psychiatric profile. Therefore, sensitivity analysis was carried out by extracting refugees and the Swedish-born with only depressive disorders from the study population and trajectories of their antidepressant use identified.

## Results

Table 1 presents the descriptive characteristics for the 2,198 refugees and the 12,199 Swedish-born youth. The majority of the refugee population resettled to Sweden ‘on humanitarian grounds’ (40%), one-fourth were ‘in need of protection’ (26%), and nearly 30% moved to Sweden as ‘family members for reunification’ to a refugee (data not shown). Compared to Swedish-born, refugees were somewhat more likely to have a lower educational level and to be married or cohabiting (Table 1). With respect to healthcare utilization during the period 2009–11, refugees sought less healthcare for most CMDs, with the exception for posttraumatic and other stress-related mental disorders. Additionally, a larger proportion of the young refugees had comorbid mental disorder along with CMD than their Swedish-born peers (71% vs 57%, respectively). However, refugees used less antidepressants (50% vs 75%, respectively), and they were also less sickness absent (7% vs. 17%) or on disability pension (7% vs 11%, respectively) than youth born in Sweden during 2009–11.

### Comparison of antidepressant use trajectories in refugees and Swedish-born

**Fig. 1:**
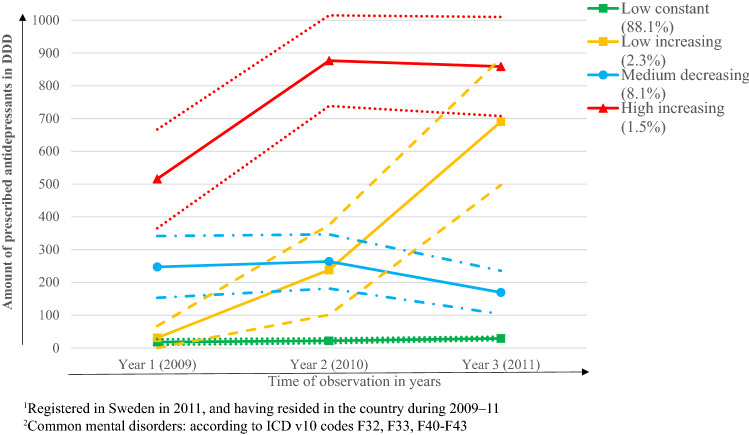
The trajectories of prescribed antidepressants among refugees^1^, aged 16–25 years, with in- or specialised outpatient care due to common mental disorder^2^ during 2009–11 (*n* = 2198)

Among the 2198 young refugees with treated CMD, the trajectory analysis identified four user groups of antidepressants, labelled ‘low constant’, ‘low increasing’, ‘medium decreasing’ and ‘high increasing’ (Fig. 1). The ‘low constant’ group (88%) was dispensed less than 100 DDDs between 2009 and 2011. The ‘low increasing’ group (2%) used less than 100 DDDs of antidepressants in 2009, which increased to around 710 in 2011. Antidepressant use decreased from around 300 DDDs to around 170 DDDs from 2009 to 2011 in the ‘medium decreasing’ group (8%), while the ‘high increasing’ group (2%) used around 500 DDDs in 2009 and up to 860 DDDs in 2011.

**Fig. 2:**
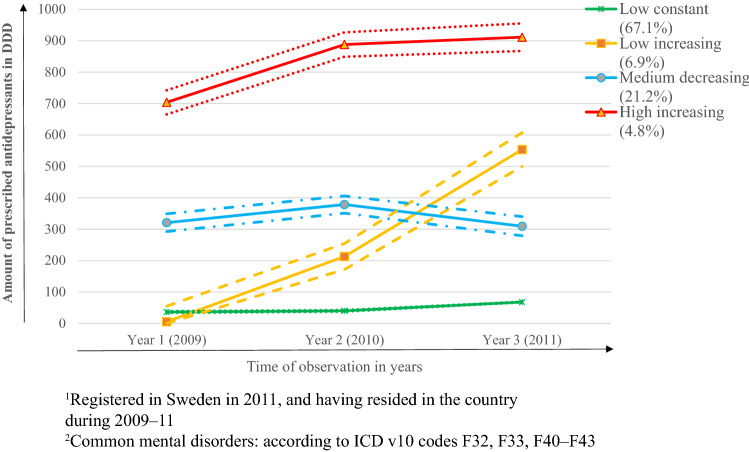
The trajectories of prescribed antidepressants among Swedish-born^1^, aged 16–25 years, with in- or specialised outpatient care due to common mental disorder^2^ during 2009–11 (*n* = 12,199)

The identified patterns of antidepressant use in Swedish-born were similar to refugees’; however, the proportions differed considerably. The proportions for ‘low constant’, ‘low increasing’, ‘medium decreasing’ and ‘high increasing’ groups for Swedish-born were 67%, 7%, 21% and 5%, respectively (Fig. 2). In the ‘medium decreasing’ group of the Swedish-born, unlike among the refugees, the decline in antidepressant level was not steep. Greatest differences in the levels of DDDs were in the high increasing group, increasing from 700 to 900 in the Swedish-born.

### Sociodemographic and medical factors discriminating trajectory groups of antidepressant use

The sociodemographic and clinical profile in the four identified trajectory groups differed considerably in the young refugees. Compared to the other antidepressant trajectory groups, refugees in the ‘low constant’ group were most often women (54%), younger (16–19 years, 25%) and with lower education level (46%). Moreover, refugees in this group were more often living with partner or parents (50%), had shorter duration of stay in Sweden (< 10 years, 50%) and being least on sickness absence (6%), on disability pension (6%) or with any mental or somatic comorbidity (Table 2). The strongest differences were observed between the ‘low constant’ and the ‘high increasing’ groups. The latter included primarily male (64%) and older individuals (20–25 years, 94%), more often with high school education (10–12 years, 48.5%) and a longer duration of stay in Sweden (> 15 years, 72.7%). Furthermore, compared to all other groups, refugees in the ‘high increasing’ group had most often sickness absence (21%), disability pension (18%), comorbid ADHD (15%), substance use (21%) and other mental disorders (54%) (Table 2). Also young refugees in the two remaining trajectory groups differed in some aspects from their peers in the other groups: those in the ‘low increasing’ group had most often a high educational level (20%), and both comorbid somatic disorders (76%) and suicidal behaviour (18%), while individuals in the ‘medium decreasing’ group were most often living in villages (22%), with 11–15 years of stay in Sweden (17%) and with a comorbid personality disorder (11%).

**Table 2:** Distributions and associations of sociodemographic and medical characteristics by trajectory group of prescribed antidepressants in refugees^1^, aged 16–25, with inpatient or specialised outpatient care due to common mental disorder^2﻿^ during 2009–11 (*n* = 2198)

Characteristics	Low constant	Low increasing	Medium decreasing	High increasing	Pearson’s Chi-Square (*p* value)	Log-likelihood test Chi-Square (*p* value)^g^	**Diff. in R**^2 *^
	*n* (%) 1936 (88.1)	*n* (%) 50 (2.3)	*n* (%) 179 (8.1)	*n* (%) 33 (1.5)			
Sociodemographic characteristics							
Sex^a^							
Female	1047 (54.1)	25 (50.0)	85 (47.5)	12 (36.4)	6.9 (0.0.8)	6.4 (0.09)	0.004
Male	889 (45.9)	25 (50.0)	94 (52.5)	21 (63.6)			
Age^a^							
16–19	486 (25.1)	8 (16.0)	22 (12.3)	< 8^¤^ (6.1)	22.4 (0.00)	0.8 (0.86)	0.000
20–25	1450 (74.9)	42 (84.0)	157 (87.7)	31 (93.9)			
Education (years)^a^							
Compulsory (≤ 9)	899 (46.4)	11 (22.0)	73 (40.8)	12 (36.4)	24.9 (0.00)	16.1 (0.07)	0.011
High school (10–12)	618 (31.9)	23 (46.0)	67 (37.4)	16 (48.5)			
University (> 12)	191 (9.9)	10 (20.0)	25 (14.0)	< 8^¤^ (9.1)			
Missing	228 (11.8)	6 (12.0)	14 (7.8)	< 8^¤^ (6.1)			
Living area^3,a^							
Big cities	823 (42.5)	20 (40.0)	67 (37.4)	13 (39.4)	2.3 (0.9)	5.0 (0.55)	0.003
Medium sized cities	747 (38.6)	21 (42.0)	73 (40.8)	14 (42.4)			
Small towns/villages	366 (18.9)	9 (18.0)	39 (21.8)	< 8^¤^ (18.2)			
Family situation^a^							
Married or cohabiting	241 (12.4)	< 8^¤^ (12.0)	19 (10.6)	< 8^¤^ (9.1)	19.3 (0.00)	4.8 (0.57)	0.003
Single^4^	1289 (66.6)	35 (70.0)	142 (79.3)	28 (84.8)			
Living with parents, < 20 years old	406 (21.0)	9 (18.0)	18 (10.1)	< 8^¤^ (6.1)			
Duration of stay in the host country (Sweden)^b^							
0–5 years	572 (29.5)	< 8^¤^ (14.0)	29 (16.2)	< 8^¤^ (9.1)	53.8 (0.00)	19.2 (0.02)	0.013
6–10 years	397 (20.5)	< 8^¤^ (10.0)	36 (20.1)	< 8^¤^ (12.1)			
11–15 years	308 (15.9)	< 8^¤^ (14.0)	30 (16.8)	< 8^¤^ (6.1)			
> 15 years	659 (34.0)	31 (62.0)	84 (46.9)	24 (72.7)			
Sickness absence during 2009–11 (in net days)							
1–90 annual net days	64 (3.3)	< 8^¤^ (6.0)	8 (4,5)	< 8^¤^ (6.1)	24.5 (0.00)	8.4 (0.21)	0.006
> 90 annual net days	58 (3.0)	< 8^¤^ (10.0)	8 (4.5)	< 8^¤^ (15.2)			
None	1814 (93.7)	42 (84.0)	163 (91.1)	26 (78.8)			
Disability pension during 2009–11							
Yes	112 (5.8)	< 8^¤^ (8.0)	30 (16.8)	< 8^¤^ (18.2)	37.4 (0.00)	10.0 (0.02)	0.007
No	1824 (94.2)	46 (92.0)	149 (83.2)	27 (81.8)			
Comorbid somatic disorders^5^ during 2009–11							
Yes	1321 (68.2)	38 (76.0)	130 (72.6)	24 (72.7)	3.0 (0.4)	1.2 (0.76)	0.001
No	615 (31.8)	12 (24.0)	49 (27.4)	9 (27.3)			
Comorbid mental disorders stratified, during 2009–11							
Personality disorder	90 (4.6)	< 8^¤^ (6.0)	19 (10.6)	< 8^¤^ (6.1)	12.0 (0.01)	2.8 (0.42)	0.002
ADHD^6^	74 (3.8)	< 8^¤^ (10.0)	12 (6.7)	< 8^¤^ (15.2)	16.7 (0.00)	1.6 (0.66)	0.001
Substance use	163 (8.4)	8 (16.0)	30 (16.8)	< 8^¤^ (21.2)	21.4 (0.00)	0.4 (0.93)	0.000
Suicidal behaviour	223 (11.5)	9 (18.0)	22 (12.3)	< 8^¤^ (12.1)	2.04 (0.56)	1.6 (0.66)	0.001
Other mental disorders	511 (26.4)	23 (46.0)	80 (44.7)	18 (54.5)	45.4 (0.00)	13.6 (0.00)	0.009

^*^Difference in Nagelkerke pseudo *R*^2^ between model including tested variable and without tested variable. Nagelkerke pseudo *R*^2^ for full model is 0.165

^¤^Numbers below 8 were expressed as ‘ < 8’ to mitigate any possibility for backward identification

^a^Measured on 31-December-2011

^b^Measured during 2009–11, based on the earliest year of inclusion in STATIV

^1^Registered in Sweden in 2011, and having resided in the country during 2009–11

^2^Common mental disorders: according to International Classification of Diseases version 10 codes F32, F33, F40-F43

^3^Type of residential area: big cities—Stockholm, Gothenburg and, Malmö; medium-sized cities—cities with more than 90,000 inhabitants within 30 km distance from the center of the city; small cities/villages

^4^Single includes divorced/separated/widowed

^5^Somatic disorders: according to ICD v10 codes all diagnoses other than F00-99, O00-99, P00-96, Q00-99, R00-99, U00-U85, V01-Y98 (except X60-84 and Y10-34)

^6^Attention deficit hyperactivity disorder

Characteristics that were significantly associated with antidepressant trajectory groups in the crude model among refugees were age, education, family situation, duration of stay in the host country, sickness absence, disability pension, comorbid personality disorder, ADHD, substance use and ‘other’ mental disorders. In the multivariate analyses, the full model for the refugees explained nearly 17% of the variance between the trajectory groups (pseudo-*R*^2^ = 0.165), whereas duration of stay in the host country (*R*^2^ = 0.013), ‘other’ comorbid mental disorders (*R*^2^ = 0.09) and granted disability pension (*R*^2^ = 0.07) significantly discriminated (*p* < 0.05) between the trajectory groups according to the Log-likelihood test.

The characteristics of the Swedish-born differed across the trajectory groups as well; however, the different profiles did not entirely match the profiles in refugee trajectory groups. Young individuals born in Sweden belonging to the ‘low constant’ group of antidepressant use were commonly low educated (41%) and with least sickness absence (15%), disability pension (8%) or any comorbid mental disorder. (Table 3). Most obvious differences to the distribution in the ‘low constant’ group were found for the Swedish-born in the ‘high increasing’ group, who were older (20–25 years, 85%), living single (74%) and in big cities (48%), having sickness absence (22%), disability pension (28%) or a comorbid personality disorder (16%), ADHD (21%) or ‘other’ mental disorders (58%) (Table 3). Finally, while Swedish-born in ‘low increasing’ antidepressant use group were mostly younger (28%), living in small town (17%) with their parents (33%) and having university education (18%), those in the ‘medium decreasing’ group commonly had high-school education (45%), lived in medium sized cities (42%) with comorbid somatic (65%) or substance use (17%) or suicidal behaviour (14%).

**Table 3:** Distributions and associations of sociodemographic and medical characteristics by trajectory group of prescribed antidepressants in Swedish-born^1^, aged 16–25, with inpatient or specialised outpatient care due to common mental disorder^2^ during 2009–11 (*n* = 12,199)

Characteristics	Low constant	Low increasing	Medium decreasing	High increasing	Pearson’s Chi-Square (*p* value)	Log-likelihood test Chi-Square (*p* value)^g^	**Diff. in** **R**^2 *^
	*n* (%) 8184 (67.1)	*n* (%) 844 (6.9)	*n* (%) 2588 (21.2)	*n* (%) 583 (4.8)			
Sociodemographic characteristics							
Sex^a^							
Women	4937 (60.3)	564 (66.8)	1734 (67.0)	368 (63.1)	45.5 (0.00)	37.4 (0.00)	0.003
Men	3247 (39.7)	280 (33.2)	854 (33.0)	215 (36.9)			
Age (in years)^a^							
16–19	2105 (25.7)	238 (28.2)	425 (16.4)	89 (15.3)	127.2 (0.00)	23.7 (0.00)	0.002
20–25	6079 (74.3)	606 (71.8)	2163 (83.6)	494 (84.7)			
Education (years)^a^							
Compulsory (≤ 9)	3394 (41.5)	312 (37.0)	928 (35.9)	228 (39.1)	60.8 (0.00)	87.4 (0.00)	0.008
High school (10–12)	3588 (43.8)	355 (42.1)	1161 (44.9)	248 (42.5)			
University (> 12)	1041 (12.7)	158 (18.7)	439 (17.0)	95 (16.3)			
Missing	161 (2.0)	19 (2.3)	60 (2.3)	12 (2.1)			
Living area^3,a^							
Big cities	3504 (42.8)	373 (44.2)	1103 (42.6)	280 (48.0)	12.6 (0.05)	13.7 (0.03)	0.001
Medium sized cities	3355 (41.0)	327 (38.7)	1080 (41.7)	234 (40.1)			
Small towns/villages	1325 (16.2)	144 (17.1)	405 (15.6)	69 (11.8)			
Family situation^a^							
Married or cohabiting	416 (5.1)	33 (3.9)	141 (5.4)	23 (3.9)	92.5 (0.00)	13.8 (0.03)	0.001
Single^4^	5324 (65.1)	532 (63.0)	1887 (72.9)	433 (74.3)			
Living with parents, < 20 years old	2444 (29.9)	279 (33.1)	560 (21.6)	127 (21.8)			
Sickness absence during 2009–11 (in net days)							
1–90 annual net days	702 (8.6)	64 (7.6)	200 (7.7)	46 (7.9)	104.9 (0.00)	77.3 (0.00)	0.007
> 90 annual net days	532 (6.5)	103 (12.2)	291 (11.2)	80 (13.7)			
None	6950 (84.9)	677 (80.2)	2097 (81.0)	457 (78.4)			
Disability pension during 2009–11							
Yes	642 (7.8)	73 (8.6)	484 (18.7)	163 (28.0)	410.4 (0.00)	184.9 (0.00)	0.017
No	7542 (92.2)	771 (91.4)	2104 (81.3)	420 (72.0)			
Comorbid somatic disorders^5^ during 2009–11							
Yes	5204 (63.6)	549 (65.0)	1683 (65.0)	356 (61.1)	4.3 (0.23)	12.7 (0.01)	0.001
No	2980 (36.4)	295 (35.0)	905 (35.0)	227 (38.9)			
Comorbid mental disorders stratified, during 2009–11							
Personality disorder	526 (6.4)	78 (9.2)	385 (14.9)	95 (16.3)	215.5 (0.00)	24.2 (0.00)	0.002
ADHD^6^	912 (11.1)	99 (11.7)	465 (18.0)	123 (21.1)	115.5 (0.00)	22.8 (0.00)	0.002
Substance use	1179 (14.4)	125 (14.8)	435 (16.8)	93 (16.0)	9.3 (0.03)	19.6 (0.00)	0.002
Suicidal behaviour	738 (9.0)	108 (12.8)	373 (14.4)	83 (14.2)	73.2 (0.00)	25.5 (0.00)	0.002
Other mental disorders	3076 (37.6)	391 (46.3)	1402 (54.2)	341 (58.5)	290.7 (0.00)	85.8 (0.00)	0.008

*Difference in Nagelkerke pseudo R^2^ between model including tested variable and without tested variable. Nagelkerke pseudo R^2^ for full model is 0.196

^a^Measured on 31-December-2011

^1^Having resided in Sweden during 2009-11

^2^Common mental disorders: according to International Classification of Diseases version 10 codes F32, F33, F40-F43

^3^Type of residential area: big cities—Stockholm, Gothenburg and, Malmö; medium-sized cities—cities with more than 90,000 inhabitants within 30 km distance from the center of the city; small cities/villages

^4^Single includes divorced/separated/widowed

^5^Somatic disorders: according to ICD v10 codes all diagnoses other than F00–99, O00–99, P00–96, Q00–99, R00–99, U00–U85, V01–Y98 (except X60–84 and Y10–34)

^6^Attention deficit hyperactivity disorder

In Swedish-born, the full model of the multivariate analyses explained nearly 20% of the variance across the trajectory groups (pseudo-*R*^2^ = 0.196), while education (*R*^2^ = 0.008), sickness absence (*R*^2^ = 0.007), disability pension (*R*^2^ = 0.017) and ‘other’ comorbid mental disorders (*R*^2^ = 0.008) contributed most in the final model according to differences in pseudo-R^2^ and were also significantly discriminating between the trajectory groups (*p* < 0.05) according to the Log-likelihood test.

### Sensitivity analysis

Three groups of antidepressant use were identified among young refugees with depressive disorders (*n* = 793) with following proportions and DDD levels during 2009–2011: ‘low constant’ (89%, 50–65), ‘medium increasing’ (10%, 225–375) and ‘medium increasing’ (1%, 240–1260). Trajectory groups for Swedish-born (*n* = 5850) were ‘low increasing’ (74%, 60–120), ‘medium increasing’ (24%, 330–470) and ‘high increasing’ (2%, 880–1075).

## Discussion

### Main findings

We found four trajectory groups of antidepressant use: ‘low constant’, ‘low increasing’, ‘medium decreasing’ and ‘high increasing’ among both young refugees and their Swedish-born peers. Still, the proportions of individuals belonging to these groups and in part the related DDD levels differed in the two populations. The proportion of refugees in the ‘low constant’ group was considerably higher than Swedish-born (88% vs 67%), respectively, whereas in the ‘medium decreasing’ group similar proportion was substantially lower (8% vs 21%). The sociodemographic and medical profile of refugees in the different trajectory groups had both similarities and differences to the profile of the Swedish-born. The most influential factors that significantly discriminated trajectory groups of antidepressant use among refugees were duration of stay in Sweden, ‘other’ mental disorders and granted disability pension, while receipt of disability pension, comorbid ‘other’ mental disorders and educational level were the most important determinants significantly discriminating trajectory groups among Swedish-born youth.

### Possible explanations of the findings

We found that 88% of young refugees had low constant levels of antidepressants over a 3-year observation period, compared to 67% of their Swedish-born peers. Moreover, 8% of the refugees and 21% and Swedish-born belonged to the ‘medium decreasing’ trajectory group. While studies on patterns of antidepressant use in young refugees are not available for comparative purpose, previous research on mental health among refugees residing in the Nordic countries suggests that they tend to have less frequent psychiatric medication use compared to the population in the host country [10, 12, 23, 39]. The difference might be explained by the fact that refugees tend to underutilize mental healthcare services and have a lack of knowledge regarding mental diseases and negative perceptions about their treatment with psychiatric medication [40–42]. Here, beliefs about antidepressants affecting negatively on personality or leading to addiction might also contribute to a lower use of such medication [43]. In addition, communication difficulties caused by language barriers, and lack of knowledge regarding the access to healthcare services in the host country may explain lower antidepressant use in refugees compared to Swedish-born [44, 45]. Finally, a lack of transcultural approach in psychiatry might contribute to lower use of antidepressants in refugees [20]. The commonly used diagnostic tools for diagnosis of psychiatric disorders, i.e., ICD codes, often lack the cultural aspect of psychiatry [46]. It is not unlikely that due to the lack of cultural competence of the healthcare professionals, the clinical presentation is misinterpreted leading to under or misdiagnosis [12, 47] and thereby delayed initiation of antidepressants, or they were prescribed other psychiatric medication than antidepressants [13]. Of course, also the lower income of refugees compared to the majority population in a high-income host country might potentially lead to lower dispensed rates of antidepressants among refugees. For instance, a study from Denmark reported that antidepressant use among persons with depressive disorders was affected by their low income [48]. However, considering the largely tax-funded healthcare system and subsidised medications in Sweden, the lower income of refugees is unlikely to be the main reason for lower antidepressant use in refugees compared to Swedish-born.

Our results show that the increase of DDDs in the high increasing antidepressant use group among refugees was two times higher than among the Swedish-born. This group comprised few individuals and was characterised by a high load of comorbidities and work disability in both refugees and Swedish-born, suggesting a higher medical severity of the underlying CMD. The relatively higher increase in antidepressant use in refugees during the observation period might be explained by a delay in seeking healthcare leading to a high medical severity at treatment onset and with time necessitating higher levels of antidepressant treatment.

We also found that the duration of stay in the host country best explained the differences across the trajectory groups of antidepressant use among young refugees. Here, a longer duration of stay in Sweden was associated with being in the ‘high increasing’ antidepressant use group and a shorter duration with the ‘low constant’ group. This is in line with a previous study on young refugees with CMDs showing that shorter duration of stay in Sweden was associated with a lower likelihood of initiating antidepressants [13]. Duration of stay in a host country plays a vital role in achieving better language skills, acculturation and understanding of the healthcare system [49, 50] that can improve health-seeking behaviour, better acceptance of antidepressant treatment and communication with the ‘healthcare personnel’. In line with this, increases of healthcare due to CMD with rising duration of stay in the host country was also shown in another recent study [9].

There were some differences in the distribution of gender, living area, family situation and education between the refugees and the Swedish-born. These differences might have affected the results related to the trajectory groups. However, our analyses showed that gender, living area and family situation had a marginal influence in discriminating between trajectory groups in both refugees and Swedish-born. Educational status had a stronger effect in these analyses. The level of education was a determinant discriminating the different trajectory groups among both the refugees and Swedish-born, though this was not significant for refugees in the multivariate analyses. Therefore, it is unlikely that these differences contributed considerably to the large differences in trajectory groups in refugees and Swedish-born. For example, low constant antidepressant use was more common among individuals with lower education. This is in line with a systematic review that concluded that low educational level and low income are associated with a poor adherence to antidepressants [51]. This association might be related to problems accessing specialised healthcare and receiving treatment as well as lack of social support, which might in itself worsen the prognosis by aggravating the depressive symptomatology.

Moreover, work disability was a discriminating factor between the trajectory groups in both Swedish born and refugees, i.e. granted disability pension occurred most frequently in individuals belonging to the ‘high increasing’ group. Granting of disability pension is a long process involving medical evaluations and work-capacity assessments and might here reflect both the medical severity and the social consequences of the underlying CMD diagnoses. The strength of discrimination related to granted disability pension between groups based on the pseudo-*R*^2^ differences, was considerably higher in Swedish-born young popuation than in young refugees. This might in part be related with the eligibility criteria of being granted disability pension and refugees having less knowledge about the social insurance system in Sweden [52].

Substance use, personality disorders, ADHD and suicidal behaviour were frequently comorbid in both young refugees and Swedish-born with CMDs, ranging from 4 to 21% in the different trajectory groups with some differences in prevalence rates related to refugee status. Most discriminating between trajectory groups were, however, other (than those mentioned above) comorbid mental disorders in both young refugees and Swedish-born. These were very prevalent across all trajectory groups in both refuges (26–54%) and Swedish-born (38–59%). These disorders might be treated with antidepressants themselves or might aggravate the severity of the underling CMD diagnosis to an extent necessitating higher antidepressant dosages.

The CMD profile in the refugees and the Swedish-born is worth discussing. Specific CMD diagnoses differed in refugees and Swedish-born, i.e., the refugees were to a larger extent affected by PTSD and other stress related disorders, whereas the Swedish-born were by depressive and anxiety disorders. PTSD and stress-related mental disorders are less likely to be treated with antidepressants, hence part of the differences in trajectory groups between refugees and Swedish-born might be due to these discrepancies. However, sensitivity analyses including individuals with depressive disorders only regarding trajectories of antidepressant use revealed similar findings in the refugees and Swedish-born as for the analyses including all CMD diagnoses.

### Strengths and limitations

To the best of our knowledge this is the first study exploring trajectory groups of antidepressant use and factors associated with such groups among young refugees and their counterparts born in the host country. The major strength of this study is the unique data with high quality, including a large population of young refugees and information on a wide range of sociodemographic and medical characteristics. Furthermore, we had the possibility to observe levels and directions of antidepressant use with detailed information on DDDs over three years with practically no loss to follow-up.

There are also some limitations worth mentioning. Both CMDs and comorbid disorders (except diabetes, ADHD and substance use) have been defined by diagnosis-specific information from specialised healthcare. This way of measuring morbidity most likely captures individuals with higher medical severity. Therefore, both CMD and comorbidity might have been subjected to underreporting. Due to the known lower healthcare seeking, underreporting might even be higher in refugees [53–55]. In addition, differences in the antidepressant use trajectories between the refugees and the Swedish-born might have been partly driven by the differences in the severity of the underlying diagnosis. Unfortunately, we did not have data to measure the severity of the underlying CMD. Moreover, we did not have information on psychotherapy and could not assess differences in psychotherapeutic treatment between refugees and Swedish-born. However, due to language and cultural barriers it is unlikely that refugees would have received psychotherapy to a larger extent than their Swedish-born peers. In fact, the opposite is more reasonable, i.e., that Swedish-born not only had more antidepressant use but also use more psychotherapy. It is, therefore, important to consider psychotherapy in future studies. Moreover, there is a possibility that refugees were prescribed hypnotics, sedatives and anxiolytics rather than antidepressants to a larger extent than Swedish-born. Still, a previous study showed only small differences regarding prescribed anxiolytics, sedatives and hypnotics between refugees with CMDs resettled in Sweden and Swedish-born [13]. Also, all the assumptions were based on the amount of dispensed antidepressant, which may not be the amount that was actually used by the study population. In addition, one may argue the decision of including those with a resident permit due to ‘family reunification to refugee’ into the ‘refugee’ group, as this group does not fall under the definition of refugees by Geneva convention, neither they were granted resident permit due to ‘in need of protection’ or on ‘humanitarian ground’. Such decision was motivated by several reasons, First, United Nations High Commissioner for Refugees (UNHCR) advocates to consider the family members and dependents of a refugee as ‘refugee’ [56]. Another important reason was to be consistent with other research for the sake of comparability [13]. Additionally, our sensitivity analysis confirmed the similarity of antidepressant trajectory groups across the sub-groups of the refugees, i.e., ‘refugees defined by Geneva convention’, ‘in need of protection’, ‘humanitarian grounds’ and ‘family reunification to refugees’. Last, the refugee population included individuals with residence permit in Sweden. For this reason, the findings cannot be directly generalised to asylum seekers and refugees in low-income countries with different healthcare, social insurance and migration policies.

## Conclusions

Four similar trajectory groups of antidepressant use among both young refugees and Swedish-born youth with CMDs were found. Still, the proportions of individuals belonging to these groups and the related DDD levels differed in the two populations. The strongest difference was in the proportions of refugees and Swedish-born in the ‘low constant’ groups of antidepressant use, which were considerably higher in refugees. The most influential factors that significantly discriminated groups of antidepressant use among refugees were duration of stay in Sweden, ‘other’ mental disorders and granted disability pension, while receipt of disability pension, comorbid ‘other’ mental disorders and educational level were the most important determinants significantly discriminating trajectory groups among Swedish-born youth. The lower use of antidepressants in refugees with CMDs compared to their Swedish-born peers calls for the need of health literacy programs for refugees and training in transcultural psychiatry for healthcare professionals.

## Data Availability

The register data used in this study contain sensitive information at an individual level and, therefore, are not publicly available due to confidentiality.
